# Accelerated Solvent Extractions (ASE) of *Mitragyna speciosa* Korth. (Kratom) Leaves: Evaluation of Its Cytotoxicity and Antinociceptive Activity

**DOI:** 10.3390/molecules26123704

**Published:** 2021-06-17

**Authors:** Yong Sean Goh, Thiruventhan Karunakaran, Vikneswaran Murugaiyah, Rameshkumar Santhanam, Mohamad Hafizi Abu Bakar, Surash Ramanathan

**Affiliations:** 1 Centre for Drug Research, Universiti Sains Malaysia, Gelugor 11800 USM, Pulau Pinang, Malaysia; gysean08@gmail.com (Y.S.G.); srama@usm.my (S.R.); 2 School of Chemical Sciences, Universiti Sains Malaysia, Gelugor 11800 USM, Pulau Pinang, Malaysia; 3 School of Pharmaceutical Sciences, Universiti Sains Malaysia, Gelugor 11800 USM, Pulau Pinang, Malaysia; vicky@usm.my; 4 BioSES Research Interest Group, Faculty of Science and Marine Environment, Universiti MalaysiaTerengganu, Kuala Nerus 21030, Terengganu, Malaysia; ramesh@umt.edu.my; 5 Bioprocess Technology Division, School of Industrial Technology, Universiti Sains Malaysia, Gelugor 11800 USM, Penang, Malaysia; mhafizi88@usm.my

**Keywords:** *Mitragyna speciosa*, accelerated solvent extraction (ASE), mitragynine, cytotoxicity, antinociceptive

## Abstract

*Mitragyna speciosa* Korth (kratom) is known for its psychoactive and analgesic properties. Mitragynine is the primary constituent present in kratom leaves. This study highlights the utilisation of the green accelerated solvent extraction technique to produce a better, non-toxic and antinociceptive active botanical extract of kratom. ASE *M. speciosa* extract had a dry yield (0.53–2.91 g) and showed a constant mitragynine content (6.53–7.19%) when extracted with organic solvents of different polarities. It only requires a shorter extraction time (5 min) and a reduced amount of solvents (less than 100 mL). A substantial amount of total phenolic (407.83 ± 2.50 GAE mg/g and flavonoids (194.00 ± 5.00 QE mg/g) were found in ASE kratom ethanol extract. The MTT test indicated that the ASE kratom ethanolic leaf extract is non-cytotoxic towards HEK-293 and HeLa Chang liver cells. In mice, ASE kratom ethanolic extract (200 mg/kg) demonstrated a better antinociceptive effect compared to methanol and ethyl acetate leaf extracts. The presence of bioactive indole alkaloids and flavonols such as mitragynine, paynantheine, quercetin, and rutin in ASE kratom ethanolic leaf extract was detected using UHPLC-ESI-QTOF-MS/MS analysis supports its antinociceptive properties. ASE ethanolic leaf extract offers a better, safe, and cost-effective choice of test botanical extract for further preclinical studies.

## 1. Introduction

*Mitragyna speciosa* Korth (Rubiaceae) is popularly known as kratom in Thailand and ketum or biak biak in Malaysia. It is traditionally used by locals in Thailand and Malaysian to relieve pain, tiredness and to treat opioid addiction [1]. *M. speciosa* leaf is reported to have cocaine-like stimulant effects at a smaller dose, whereas at higher dosage, it possesses opioid-like sedative narcotic effects [2,3]. Previous phytochemical studies in *M. speciosa* have reported the presence of bioactive secondary metabolites from the phytochemical groups such as indole alkaloids, flavonoids, triterpenoids, saponins, and glycoside [4]. This plant has been reported for various biological and pharmacological properties through preclinical studies such as antioxidant, antibacterial, antiproliferative, anti-inflammatory, and antinociceptive [5,6].

It was previously reported that conventional solvent extraction methods use a large amount of toxic organic solvents (MeOH & CHCl3), are labour intense, and have longer extraction times and low extraction yields [7]. These problems can be overcome by using modern, advanced, and green extraction techniques developed in agreement with Green Analytical Chemistry [8]. The systems can also be fairly automated in different manners [9]. Accelerated solvent extraction (ASE) is also considered to be such a technique. ASE is an automated rapid-extraction technique, which using elevated pressure to increase the contact area with analyte by forcing the solvent into the pores of the sample matrix, thus giving better analyte recovery [10,11]. Based on the previous reports, the ASE approach is economic, less time-consuming, has low solvent usage, and produces high extraction yield [12]. In the current field of extraction technology, the ASE technique was recommended to be one of the best extraction techniques for various processes, which focused on herbal, food, pharmaceuticals, and nutraceutical-based research and product developments [12].

Thus, in this research, we aimed to study the extraction efficiency of the ASE technique in kratom leaves using green solvents such as water, ethanol, and ethyl acetate, which are more preferred by industries due to their low toxicity, safeness, and environmentally friendly properties with the comparison of the most commonly used extraction solvent, methanol [13,14]. The effect of the ASE technique on extraction yield, total phenolic content, total flavonoid content, cytotoxicity, and the antinociceptive activity of *M. speciosa* leaf extracts were revealed for the first time in this study. Additionally, the phytochemical profiling of the ASE *M. speciosa* leaf extracts using UHPLC-ESI-QTOF-MS/MS analysis was conducted to support its cytotoxicity and antinociceptive effects.

## 2. Results

At first, water was chosen as the extraction solvent to extract the kratom leaves using the ASE technique and to measure its extraction efficiency. Moreover, the time of extraction was chosen to be optimised in this ASE technique. However, there was no noticeable change in dry yield (2.00–2.14 g) or mitragynine content (1.54%–1.83%) after extraction with water using three different extraction times, 5, 10, and 20 min, respectively (see Table 1). For the subsequent tests on solvent extraction, the method employing a minimum extraction time of 5 min was chosen. After then, the kratom leaves were extracted using organic solvents of differing polarities, which were methanol, ethanol, and ethyl acetate, respectively (see Table 2). This resulted in the extract’s dry yield, and their respective mitragynine content ranged from 0.53 to 2.91 g and 1.83 to 7.19%. HPLC chromatograms are shown in (Figure S1, Supplementary Material). Though the percentage of dry yield varied among the extracts, the mitragynine percentage was consistent to the respective extracts. To our advantage, the ASE method required only a small amount (100 mL) of solvent to extract the kratom leaves.

The TPC and TFC for kratom leaf extracts were determined. The ASE EtOAc kratom leaf extract (459.78 ± 5.47 GAE mg/g) had the highest TPC compared to other ASE extracts (see Table 3). TFC was remarkably high in the ASE EtOH kratom leaf extract (194.00 ± 5.00 QE mg/g), compared to other ASE extracts (see Table 4).

**Figure 1:**
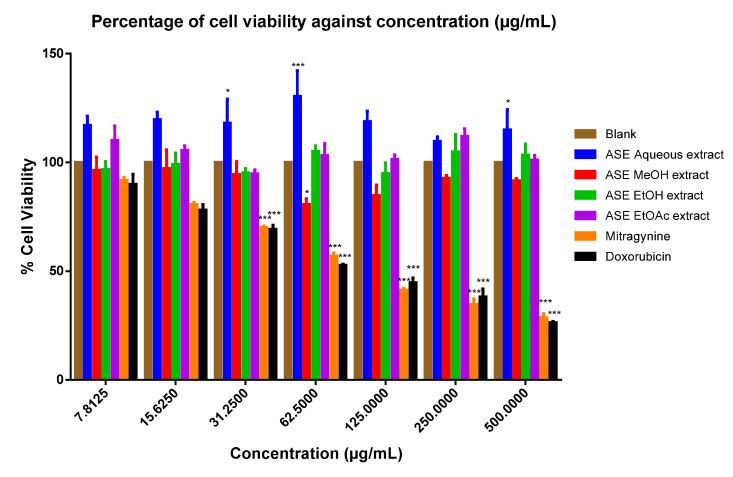
Percentage viability of human embryonic kidney cells against various concentrations of treatment with mitragynine, doxorubicin, and *M. speciosa* ASE leaf extracts. Data shown as mean ± SEM (*n* = 6). * *p* < 0.05, *** *p* < 0.001 compared with DMSO as blank (one-way ANOVA, followed by Dunnett’s test).

**Figure 2:**
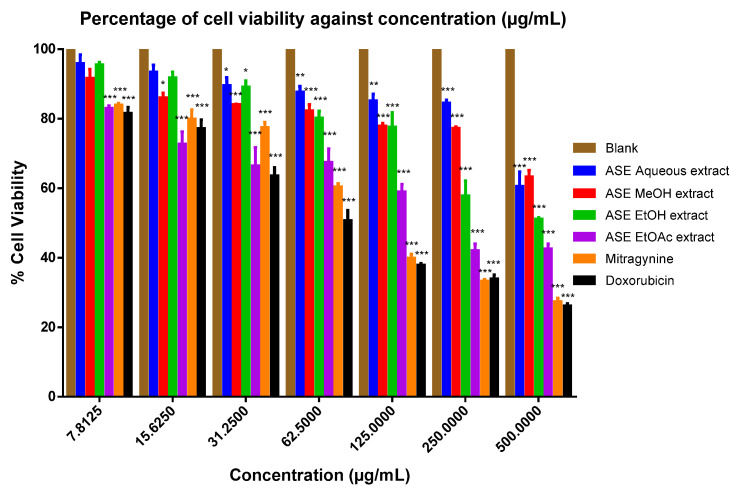
Percentage viability of HeLa Chang liver cells against various concentrations of treatment with mitragynine, doxorubicin, and *M. speciosa* ASE leaf extracts. Data shown as mean ± SEM (*n* = 6). * *p* < 0.05, ** *p* < 0.01, *** *p* < 0.001 compared with DMSO as blank (one-way ANOVA, followed by Dunnett’s test).

When cytotoxicity was assessed on respective tested cell lines, with exception to the ASE EtOAc kratom leaf extract, the ASE MEOH and EtOH kratom leaf extracts showed higher IC_50_ values against HEK-293 kidney (IC_50_ > 500 μg/mL) and HeLa Chang liver (IC_50_ > 500 μg/mL) cell lines. However, the mitragynine standard had lower IC_50_ (HEK-293 kidney cell; IC_50_:112.30 ± 17.59 μM; HeLa Chang liver cell; IC_50_: 210.04 ± 0.80 μM) (see Figure 1 and Figure 2) (Supplementary Material, Tables S1 and S2). Table 5 provides a summary of IC_50_ values for the ASE kratom leaf extracts, respectively. Thus, all of the ASE kratom leaf extracts were found to be nontoxic (IC_50_ > 500 ug/mL) towards HEK-293 and HeLa Chang liver cell lines.

**Figure 3:**
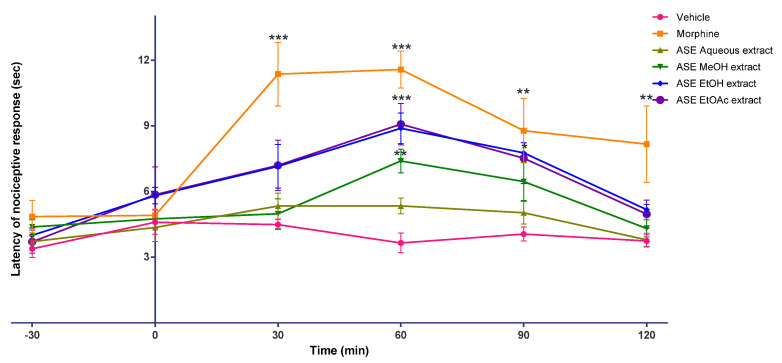
Effects of vehicle, morphine, and *M. speciosa* ASE leaf extracts on Swiss albino mice nociceptive response in a hot plate test. Extracts were administrated orally (morphine injection, s.c.), and nociceptive response was measured at 30 min interval over 120 min in mice. Data shown as mean ± SEM (*n* = 6). * *p* < 0.05, ** *p* < 0.01, *** *p* < 0.001 compared with vehicle group (one-way ANOVA, followed by Dunnett’s test).

**Figure 4:**
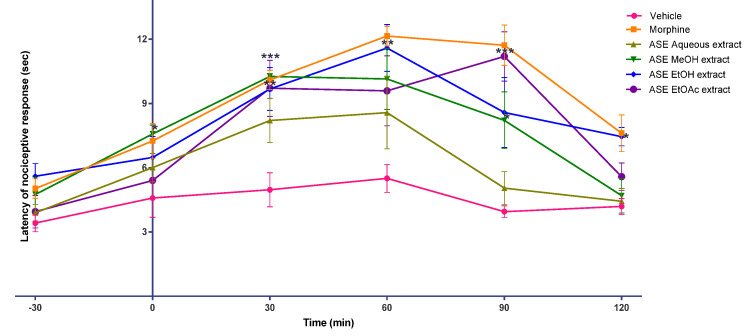
Effects of vehicle, morphine and *M. speciosa* ASE leaf extracts on Swiss albino mice nociceptive response to tail-flick test. Extracts were administrated orally (morphine injection, s.c.), and nociceptive response was measured at 30 min interval over 120 min in mice. Data shown as mean ± SEM (*n* = 6). * *p* < 0.05, ** *p* < 0.01, *** *p* < 0.001 compared with vehicle group (one-way ANOVA, followed by Dunnett’s test).

The analgesic activity of the ASE kratom leaf extracts was tested in male Swiss albino mice using hot plate and tail-flick tests. From the hot plate and tail-flick tests (see Figure 3 and Figure 4), after the onset of action, the antinociceptive effect of morphine persisted throughout the study in all mice treated with morphine (30 min, to 120 min; peaked at 60 min). Interestingly, the antinociceptive effect of the ASE EtOH kratom leaf extract (200 mg/kg) was comparable to that of morphine-treated mice, as shown in Tables S3 and S4, Supplementary Materials. They had a similar onset and time of antinociceptive action with morphine. However, in a hot plate test, the ASE EtOH kratom leaf extract (200 mg/kg) showed a delayed start of action (60 min) and a short duration of antinociceptive effect (30 min). Nevertheless, both the ASE EtOAc and MeOH extracts kratom leaf showed an intermittent onset and duration of antinociceptive action in both the hot plate and the tail-flick tests.

A total of 19 phytochemicals were identified by UHPLC-ESI-QTOF-MS/MS chemical profiling analysis on the ASE kratom leaf extracts and matched with the available database (MassHunter Qualitative Analysis-Metlin Database). Table 6 summarises the bioactive phytochemicals, mainly indole alkaloids and flavonols. The presence and identification of these phytochemicals confirmed with the reports published in the previous literature [4,15].

## 3. Discussion

Extraction techniques, time, and type of solvents play a vital role in extracting targeted and non-targeted phytochemicals from plants [23,24]. For the first time, the accelerated solvent extraction (ASE) technique was employed to extract the kratom leaves. The extraction efficiency in terms of dry yield and mitragynine content was compared using various extraction solvents. There are other extraction methods available and frequently used to extract plant materials, such as Soxhlet, maceration, and ultrasonic-assisted extraction techniques. However, these methods have their own limitations and constraints in terms of extraction efficiency, duration, and volume of solvent used for extraction. Ultrasonic-assisted extraction technique may yield good results within a short time frame; yet, since it uses a frequency in the range of 20 kHz to 2000 kHz, it may cause undesirable modification in the structure of the compounds [25]. Soxhlet and maceration methods would take a significantly longer time to extract plant materials. Moreover, Soxhlet involves high temperatures in extracting the plant materials, which may cause degradation of some compounds during the extraction [7,25]. Besides that, ASE is one of the preferred green extraction techniques in herbal extraction as it is is environmentally friendly, energy-efficient, and can be handled automatically. No additional filtration is required after the removal of the solvent by the ASE instrument. ASE is also highly selective and has been used in the extraction of various bioactive phytochemicals such as saponins, flavonoids, and essential oils from plants [7,26]. Furthermore, ASE techniques consume shorter extraction durations compared to the other conventional extraction techniques.

In the present study, water was first used as the extraction solvent to obtain the kratom leaf extracts. Water was chosen due to its vast usage and is preferred in the extraction protocols of traditional medicine (decoctions) and in modern herbal technologies [27]. The ASE technique on kratom leaf extraction was optimised with extraction time. No noticeable differences were observed in dry yield and mitragynine content after extraction with water for the three different extraction periods. The study revealed that the shortest extraction time for ASE was 5 min, which subsequently served as the basis for subsequent testing. ASE aqueous extract (dry yield; 2.14 g mitragynine content; 1.83%; time 5 min) yielded a substantial amount of phytoconstituents in a short duration. Solvent selection is one of the most critical steps in sample preparation for phytochemical extraction involving cosmetics, foods, and pharmaceuticals. Water is preferred as an extraction solvent for plant-based preparation (decoction) but is not suitable for extracting hydrophobic compounds with low water solubility [26]. The polarity of solvents is also a vital factor that affects the extraction yield of a plant and its phytochemical content. These, in turn, affects its biological or pharmacological activities [28]. Thus, in this study, the influence of various solvents on kratom leaves extraction was explored using different organic solvents such as MeOH, EtOH, and EtOAc apart from water. MeOH, despite its toxicity, is an extraction solvent widely used due to its ability to extract different types of phytochemicals and produce higher yields of plant extract, whereas EtOH and EtOAc are the two non-toxic and environmentally friendly solvents that are known as green solvents [13,14].

Apart from that, the ASE kratom leaf extracts were subjected to TPC and TFC tests. The TPC value was relatively higher for the ASE organic solvent {(EtOAc (459.78 ± 5.47 GAE mg/g); MeOH (448.67 ± 8.33 GAE mg/g); EtOH (407.83 ± 2.50 GAE mg/g)} extracts than the aqueous extracts (367.42 ± 3.06 GAE mg/g). Instead, in the TFC analysis, except for EtOH extract (194.00 ± 5.00 QE mg/g), both EtOAc (141.50 ± 10.00 QE mg/g) and MeOH (125.25 ± 1.25 QE mg/g) extracts were comparatively similar to the aqueous extracts (115.25 ± 6.25 QE mg/g). Previous studies have shown that aqueous plant extracts contain low levels of phenolics and flavonoids despite their higher yield, and organic extracts contain high levels of phenolics and flavonoids [26,29]. This observation is apparent in the ASE EtOH extract. It is also evident that the type of phytochemicals extracted depends primarily on the nature of the solvent used and the solubility of the compounds [30,31].

Kratom leaf extracts were further evaluated for their cytotoxicity on HeLa Chang liver and HEK-293 cell lines using MTT assay. Except for the ASE EtOAc kratom leaf extract, all the tested extracts showed IC_50_ > 500 µg/mL. The pure drug, mitragynine had considerably higher IC_50_ values (HEK-293 kidney cell; IC_50_:112.30 ± 17.59 µM; HeLa Chang liver cell 210.04 ± 0.80 µM) (see Table 5). Mitragynine in the ASE extracts constitutes in the range between 6.5% and 7.2%, which may suggest the consistency of the ASE extraction procedure in the extraction of bioactive phytochemicals. Moreover, the ASE technique using organic solvents effectively extracts mitragynine (6.5–7.2%) from its leaves in a short period of time compared to reported value by previous studies using Soxhlet (8–10%), maceration (4.7–6.24%), and ultrasound-assisted extraction (1.96%) techniques [5,32,33,34]. According to American National Cancer Institute, an IC_50_ value of less than 30 µg/mL (extracts) and 4 µg/mL (10 µM) (compound) can be considered cytotoxic against cancer cells after 48 to 72 h of treatments [35,36]. It can be generalised that the ASE extracts are non-cytotoxic towards HeLa Chang liver and HEK-293 cell lines. The results suggest that the synergistic interactions between active phytochemicals in the kratom leaf extracts could decrease the toxic effect of alkaloids [37]. Furthermore, reported that a high content of phenolics in extracts could support and increase cell survival in HEK-293 cell line.

Besides that, extracts from kratom leaves obtained using the ASE technique were evaluated for their analgesic activity using hot plate and tail-flick tests. The ASE kratom leaf extract dose was fixed at 200 mg/kg. The dose selection was based on an earlier analgesic study using the methanol extract [34]. In all morphine-treated mice, the antinociceptive effect of morphine persisted for the duration of this study (30–120 min; peaked at 60 min). Interestingly, the antinociceptive effect of the ASE EtOH extract (200 mg/kg) was similar to that of morphine in a tail-flick test. It had a similar beginning and duration of antinociceptive action with morphine-treated mice. However, in a hot plate test, the ASE EtOH kratom leaf extract (200 mg/kg) showed a delayed onset of action (60 min) and a short duration of antinociceptive effect (30 min). The ASE EtOAc and MeOH kratom leaf extracts showed variations in the onset and duration of antinociceptive action on both hot plate and tail-flick tests. No antinociceptive effect was identified for the ASE aqueous extract at the tested dose of 200 mg/kg.

Hot plate and tail-flick methods are used in animal models for pain evaluation. Thermal and radiant heat is used, respectively, in the hot plate and the tail-flick as a stimulus during the evaluation. In both pain models, the stimuli cause pain through heat-mediated injury of tissues and inflammation. These could lead to the discharge of peripheral mediators. The tail-flick test is used to study the antinociceptive activity of the spinal and primarily the spinal response. As for the tail-flick test, action is indicated by the withdrawal of the tail from the incident radiant heat [38]. The hot plate test is mainly a supraspinal response and, suggested for centrally acting drug analgesic profiling, such as opiates, since paw licking is the only behaviour affected by opioids. In a hot plate model, the commonly noted pain reflex actions are jumping and licking of paws. However, it is not advisable to be used in analgesic profiling of peripheral drugs [39,40,41]. It is important to note that the ASE EtOH kratom leaf extract (200 mg/kg) could alleviate pain in mice. Modulation of the mechanism of spinal and supraspinal pain may be responsible for this action. Moreover, it can be observed that there is better modulation of pain perception through the spinal (tail-flick) when compared to the supraspinal region (hot plate). However, this outcome warrants long-term efficacy and safety studies, but it has the medicinal potential to be developed as an effective analgesic.

UHPLC-ESI-QTOF-MS/MS phytochemical analysis and spectral matching have identified and predicted that bioactive flavonols and indole alkaloids are the major components in the ASE EtOH kratom leaf extract. *M. speciosa* (kratom) is a rich source of bioactive components, which includes the presence of more than 40 alkaloids. Besides mitragynine, speciociliatine, paynantheine, and 7-hydroxymitragynine were reported for having potent analgesic effects mediated via the *μ*-opioid receptor (MOR) [42,43,44]. Flavonoids, especially flavonol groups, have also been reported to be responsible for the antinociceptive effect, involving the central and peripheral mechanism through the hot plate and tail-flick tests [45,46]. For example, quercetin is reported to bind to the α 2-adrenergic receptors of the central and peripheral nervous systems and has a dose-dependent antinociceptive effect [15]. In addition, quercetin and its derivative, rutin, also activate the cGMP/PKG/ATP-sensitive potassium pathway and deactivate cellular regulating proteins in neurons to block pain and inflammation reactions with fewer side effects [47]. Taken together, the agonist, antagonist, synergistic interactions among alkaloids, flavonols, and other active constituents acting on multiple targets could have collectively exerted the antinociceptive effect. the ASE EtOH kratom leaf extract provides better nociceptive relief in mice compared to the ASE EtOAc and ASE MeOH kratom leaf extracts despite their similar mitragynine content; ASE MeOH (0.4314 mg), ASE EtOH (0.3918 mg), and ASE EtOAc (0.4074 mg) (see Table 2). This suggests the involvement of other bioactive phytochemicals besides mitragynine in the antinociceptive activity of the ASE EtOH kratom leaf extract. The phytochemical composition may vary in quantity, or some chemical entities may not be present in the ASE EtOAc and MeOH extracts. This accounts for the intermittent antinociceptive effect of these extracts (ASE EtOAc and ASE MeOH) in the experimental studies. The ASE aqueous extract had a low alkaloid (e.g., mitragynine:0.1098 mg) and phenolic concentrations, which might explain the absence of its antinociceptive action in mice.

## 4. Materials and Methods

The scheme for the overall summary of research activities is illustrated in Figure S2, Supplementary Materials.

### 4.1. Plant Material

Fresh leaves of *M. speciosa* (1 kg) were collected from Permatang Pauh, Penang. The GPS location is 5°22′17.0″ N 100°27′01.0″ E. The leaves were identified by botanists from the School of Biological Sciences, University Science Malaysia (USM), and the voucher specimen (No. 11074) was deposited.

### 4.2. Chemicals

HPLC grade methanol and silica gel 60 were purchased from Merck (Darmstadt, Germany), while analytical reagent grade methanol and formic acid were purchased from Fisher Scientific (Loughborough, UK). Analytical reagent grade ethyl acetate and HPLC grade acetonitrile were obtained from QReC (Asia) Chemical Co. Ltd. (Selangor, Malaysia). Analytical reagent grade ethanol and hexane were obtained from R&M Chemicals (Essex, UK). Sephadex LH-20 was purchased from Sigma-Aldrich Co. (St. Louis, Missouri, USA). Mitragynine standard (purity ≥ 99%) was purchased from ChromaDex Inc. (Los Angeles, CA, USA). Deionised water was prepared using an Elga Classic UVF ultrapure water purifier system (Elgastat, Bucks, UK). Deuterated acetone (acetone-*d*_6_) for NMR analysis was acquired from Merck (Darmstadt, Germany). In in vitro assay, molecular biology grade dimethyl sulfoxide (DMSO) was purchased from Bio Basic (Markham, Canada); eagle’s minimum essential medium was (EMEM) was purchased from American type culture collection (ATCC) (Rockville, MD, USA); 3-(4,5-dimethylthiazol-2-yl)-2,5-diphenyltetrazoliumbromide (MTT), fetal bovine serum (FBS), and phosphate-buffered saline (PBS) were acquired from Gibco/BRL Life Technologies Inc. (Eggenstein, Germany). Doxorubicin hydrochloride was purchased from EMD Chemicals (Darmstadt, Germany). For conducting TPC and TFC tests, sodium carbonate (Na_2_CO_3_), sodium nitrite (NaNO_2_), aluminium chloride hexahydrate (AlCl_3_.6H_2_O), and sodium hydroxide (NaOH) were purchased from Sigma-Aldrich Co. (St. Louis, MO, USA). Meanwhile, Folin–Ciocalteu (FC) reagent was acquired from R&M Chemicals (Essex, UK). In the animal behaviour study, 1,2-propylene glycol was obtained from R&M Chemicals (Essex, UK). Tween-80 was purchased from Sigma-Aldrich Co. (St. Louis, MO, USA), and morphine hydrochloride was purchased from Johnson Matthey (Edinburgh, UK).

### 4.3. General Instrumentations

Gradient-mode RP-HPLC was carried out using an Agilent 1200 Series HPLC System (Santa Clara, CA, USA) with a diode array detector (DAD). The system was equipped with an autosampler injector, column oven, quaternary pump, and solvent reservoirs. Chemstation LC3D software was used for data analysis. Chromatography separation was performed using Agilent Zorbax Eclipse Plus C18 (4.6 × 150 mm, 3.5 μm) column at room temperature (25 °C). The mobile phase was a mixture of solvent A: 0.1% formic acid, pH 2.99, and solvent B: acetonitrile, with a flow rate of 1.0 mL/min. The gradient program was set as 0–10.5 min, 10 % B; 10.5–12 min, 45 % B; 12–16 min, 70% B; and 16–18 min, 10% B. The total run time was 18 min with mitragynine eluting at 10.3 min. The injection volume set at 10 μL and chromatograms were monitored at a wavelength of 254 nm and given in Supplementary Materials. The mobile phase was prepared freshly daily, filtered through 0.2 µm nylon membrane filter and degassed for 10 min before injecting. The structure of isolated mitragynine was further confirmed by GC-MS on an HP 6890A GC system (Santa Clara, CA, USA) spectrometer, ^1^H-NMR and ^13^C-NMR spectra on a BRUKER ASCEND^TM^ 700 MHz spectrometer and 175 MHz spectrometer, respectively (Karlsruhe, Germany), and spectra were given in Figures S3–S5, Supplementary Materials. UHPLC model 1290 Infinity with LC-MS-QTOF model G6550A, Agilent Technologies (Santa Clara, CA, USA), with a dual AJS ESI detector, was used. It was comprised of a binary pump, a HiP sampler, and thermostatically controlled column compartment with a DAD. All samples were filtrated by a nylon filter with pore size 0.45 μm. The scanning range was from 100 to 1700 *m*/*z* for MS/MS in positive mode of ionisation. Separation was achieved through Agilent Zorbax Eclipse Plus C18 (4.6 × 100 mm, 3.5 μm). Gradient mobile phase was similar to RP-HPLC. Data interpretation was carried out using MassHunter Qualitative Analysis-Metlin Database. Resolved peaks were further identified with the assist of reported values from previous literature and shown in Table S5, Supplementary Materials [22].

### 4.4. Preparation of Extracts

Dionex accelerated solvent extractor 350 (Dionex Corporation, Sunnyvale, CA, USA) was used for the extraction of *M. speciosa* leaves following protocol Hazlina et al. in 2015 [48], with slight modifications. A 66 mL stainless steel ASE extraction cell was filled with 10 g of grinded *M. speciosa* leaves. The pre-set temperature was 60 °C, static extraction time was 5 min and ran for total of two extraction cycles. The solvents used for the extraction were water, methanol, ethanol, and ethyl acetate. Then, the cell was rinsed one time with fresh solvent and purged with nitrogen gas for 3 min. Extracts were filtered and solvent was removed and then freeze-dried into powder.

### 4.5. Isolation of the Mitragynine

Mitragynine was isolated following the procedures described by Goh et al. (2011) [49] and Jamil et al. (2013) [50], with slight modifications. *M. speciosa* leaves were washed and then put into the oven at 40 °C to dry for 3 days. The dried leaves were then made into powder using an electric grinder. About 500 g of *M. speciosa* leaves powder was extracted with Soxhlet technique using methanol as a solvent. The yield obtained from the methanol extract was about 80.5 g after rotary evaporation. After this, 10 % acetic acid solution was added to the methanol extract, and then filtered and washed with hexane. The acidic solution was separated and basified with 25% ammonia solution to pH 9. The alkaloids were then extracted with chloroform. This chloroform extract was evaporated under vacuum to produce about 4.95 g alkaloidal extract, which was then loaded into the silica column chromatography using a hexane and ethyl acetate (80:20 *v*/*v*) mobile phase. About 1.25 g of mitragynine with 94–95 % purity was obtained. The mitragynine was further purified with a column packed with Sephadex Liphophilic LH-20 using methanol as the mobile phase. The total yield of pure mitragynine was 852 mg with 98 % purity in HPLC. The ^1^H-NMR and ^13^C-NMR spectral data are consistent with the published data) [49] (see Table S1).

### 4.6. Determination of Phenolic Contents and Total Flavonoid Contents

#### 4.6.1. Total Phenolic Contents (TPC)

TPC of *M. speciosa* extract was carried out utilising the FC method from Farooq et al. (2020) [51]. One hundred microliters of extract (1 mg/mL) were prepared in 95% ethanol and mixed in 2 mL of 2 % Na_2_CO_3_ solution. After 5 min incubation, 100 μL of FC reagent was added to each sample. The mixture was then incubated in the dark for 30 min, and the absorbance was measured at 750 nm using the Multiskan Go spectrophotometer (Thermo Scientific, Waltham, MA, USA). Standard curve for TPC was constructed using gallic acid (25–800 µg/mL) with samples presented as milligram of gallic acid equivalent per gram dry extract (mg GAE/g DE).

#### 4.6.2. Total Flavonoid Contents (TFC)

Estimation of TFC in *M. speciosa* extract was evaluated according to the aluminium chloride colourimetric method reported by Farooq et al. (2020) [51], with slight modifications. Standard curve for TFC was generated using quercetin (6.25–200 µg/mL). Each sample was prepared in 95% ethanol. After that, 100 μL of the sample, 1133 µL of 30% ethanol, 50 μL of 0.5 molars of NaNO_2_ solution, and 50 μL of 0.3 molar AlCl_3_.6H_2_O solutions were mixed in a tube. The mixture was incubated in the dark for 5 min, and then 333 µL of 1 molar NaOH solution was added. The blank used consisted of the extract and all reagents except AlCl_3_.6H_2_O. Absorbance was measured at 415 nm by a Multiskan Go spectrophotometer. The results were expressed as milligrams of quercetin equivalent per gram dry extract (mg QE/g DE).

### 4.7. Preparation of Extracts and Cell Treatment

One mg of *M. speciosa* extract was weighed and dissolved in 1000 µL of EMEM to make a 1000 µg/mL stock solution. After that, the stock solution was diluted to a concentration from 7.8125 µg/mL to 500 µg/mL.

#### 4.7.1. Cell Cultures and Conditions

Human embryonic kidney (HEK-293) (ATCC CRL-1573) and HeLa Chang liver cells (ATCC CCL-13) were gifted from Professor Sharif Manshor, Centre for Drug Research, USM. HEK-293 is considered an immortalised and malignant cell. HeLa Chang liver cells were previously isolated from cervical cancer cells) [35]. For this study, both cells were cultured in EMEM supplemented with 10% FBS and 1% penicillin/streptomycin under 5% CO_2_ humidified atmosphere at 37 °C.

#### 4.7.2. MTT Cell Viability Assay

MTT assay was carried out following the protocol reported by Tohar et al. (2019) [52]. Cells were harvested at 70–80% confluency and centrifuged at 1200 rpm/25 °C for 3 min. The cells were counted under a microscope using a haemacytometer and were then seeded into 96 well plates at 1 × 10^5^ cells per mL. Incubation was set to 5% CO_2_ at 37 °C for 24 h to allow for cell attachment. After that, the unattached cells were removed carefully. Cells were then treated with 100 µL of 1 mg/mL *M. speciosa* leaves extract or compound and made up to a final volume of 200 µL, further incubated for 20 h. Twenty µL MTT reagent (5 mg/mL) was then added into each well and incubated at 5% CO_2_, 37 °C for 4 h. The mixture of cell media and MTT were removed, and the purplish formazan crystals were dissolved in dimethyl sulfoxide (DMSO), further incubated in a dark environment for 15 min at 25 °C. The absorbance was read on the Multiskan Go spectrophotometer (Thermo Scientific, Waltham, MA, USA) at 570 nm wavelength. SkanIt software 4.0 was used for data analysis. All experiments were performed in triplicate. Cell viability was determined using the formula below:

Cell Viability = (Abs_Treated cells_/Abs_Untreated cells_) × 100

### 4.8. Animal Behaviour Study

#### 4.8.1. Preparation of Extracts for Animal Behaviour Study

*M. speciosa* extracts were dissolved in a mixture of propylene glycol, Tween-80, and distilled water in a ratio of 4:1:4 (*v*/*v*/*v*). The final solution of extracts was administered as a single dose of 200 mg/kg p.o. body weight in a volume of 0.1 mL/10 g body weight. The extracts and vehicle were orally administered to the mice once the experiment started at 0 min. Morphine sulfate was used as a positive control, diluted in 0.9% saline with vehicle, and was administered subcutaneously at a dose of 5 mg/kg body weight in volume of 0.1 mL/10 g body weight.

#### 4.8.2. Animals

Male Swiss albino mice with weight ranging from 25–30 g were chosen. The animals were kept in groups of five under room temperature (25 °C) with free access to food and water provided ad libitum. The mice were acclimatised for 1 week prior to experimentation. Study protocols were reviewed and approved by the Institutional Animal Care and Use Committee (IACUC), USM (USM/IACUC/2019/(118) (995).

#### 4.8.3. Hot Plate Test

The hot plate test was evaluated according to the method described in previous studies [53,54] with slight modifications. The hot plate (IITC Life Science Inc., Woodland Hills, CA, USA) was maintained at 55 °C ± 1 °C. Six mice per group were administered *M. speciosa* ethanol extract (200 mg/kg, p.o.), ethyl acetate extract (200 mg/kg, p.o.); methanol extract (200 mg/kg, p.o.); aqueous extract (200 mg/kg, p.o.); morphine (5 mg/kg, s.c.); and vehicle (control), respectively. The latency time of nociceptive response such as jumping or licking of a hind limb was measured every 30 min over a 120 min period. The cut-off time was fixed at 45 s to prevent further tissue damage.

#### 4.8.4. Tail-flick Test

The tail-flick test was performed according to the previously described methods [34,55]. The analgesiometer (IITC Life Science Inc., Woodland Hills, CA, USA) was maintained at 45 °C ± 1 °C. Dosage of *M. speciosa* extracts, morphine, and vehicle were similar to the hot plate test with cut-off time at 10 s. The latency time of tail flicking response was measured in 30 min intervals for 120 min.

### 4.9. Statistical Analysis

Statistical analyses were used to evaluate dry yield, mitragynine content, TPC, TFC, MTT cytotoxicity, and antinociceptive results of *M. speciosa* extracts by one-way analysis of variance (ANOVA) test using GraphPad Prism software version 6.0. *p*-value lesser than 0.05 was considered significantly different.

## 5. Conclusions

A rapid ASE technique was utilised to extract kratom leaves using green solvents for its preclinical evaluation. The method produced a dry yield of (0.53–2.91 g) in a short time (5 min) with a low amount of solvent usage (<100 mL). the ASE technique produced a constant yield of mitragynine (6.53–7.19%) despite the usage of different polarity solvents in kratom leaves extraction. the ASE leaf extracts were non-cytotoxic (IC_50_ > 500 μg/mL) towards the tested cell lines, HeLa Chang liver and HEK-293. In mice, the ASE EtOH kratom leaf extract (200 mg/kg) showed an antinociceptive effect which is comparable to morphine (5 mg/kg). This study highlighted the usage of the ASE technique as a suitable solvent extraction technique in kratom leaves extraction, which could benefit the utilisation of kratom leaf extracts in preclinical and clinical assessments to support their medicinal benefits.

## Figures and Tables

**Table 1:** Dry yield and mitragynine content in *M. speciosa* ASE leaves extracted with water for 5, 10, and 20 min.

Sample	Time (min)	Mean of Dry Yield (g)	Mean Percentage of Mitragynine (%) Mean ± SEM
ASE aqueous	5	2.14 ± 0.15	1.83 ± 0.08
	10	2.01 ± 0.04	1.57 ± 0.29
	20	2.00 ± 0.29	1.54 ± 0.17

Data expressed as mean ± SEM, *n* = 3. The initial mass of the raw plant material was 10 g. Data shows no significant difference among the ASE aqueous extracts using three different extraction duration, with multiple comparisons (one-way ANOVA, followed by Tukey’s test).

**Table 2:** Dry yield and mitragynine content in *M. speciosa* ASE leaves extracted, respectively, with 100% of water, MeOH, EtOH, and EtOAc.

Sample	Time (min)	Mean of Dry Yield (g)	Mean Percentage of Mitragynine (%) Mean ± SEM
ASE aqueous	5	2.14 ± 0.15 ^b^	1.83 ± 0.08 ^a^
ASE MeOH	5	2.91 ± 0.21^c^	7.19 ± 0.30 ^b^
ASE EtOH	5	2.26 ± 0.09 ^bc^	6.53 ± 0.20 ^b^
ASE EtOAc	5	0.53 ± 0.11 ^a^	6.79 ± 0.59 ^b^

Data expressed as mean ± SEM, *n* = 3. The initial mass of the raw plant material was 10 g. Data with different alphabet superscript letters show significant difference at *p* < 0.05, among different solvents, with multiple comparisons (one-way ANOVA, followed by Tukey’s test).

**Table 3:** Total phenolic contents (as GAE) in *M. speciosa* ASE leaf extracts obtained from various extraction solvents.

Extract	Mean of Absorbance	Total Phenolic Content in Dry Extract (GAE mg/g), Mean ± SEM
ASE aqueous	0.430	367.42 ± 3.06 ^a^
ASE MeOH	0.527	448.67 ± 8.33 ^c^
ASE EtOH	0.478	407.83 ± 2.50 ^b^
ASE EtOAc	0.540	459.78 ± 5.47 ^c^

Data expressed as mean ± SEM, *n* = 3. Data with different alphabet superscript letters show significant difference at *p* < 0.05, among different solvents, with multiple comparisons (one-way ANOVA, followed by Tukey’s test).

**Table 4:** Total flavonoids content (as QE) in *M. speciosa* ASE leaf extracts obtained from various extraction solvents.

Extract	Mean of Absorbance	Total Flavonoids Content in Dry Extract (QE mg/g), Mean ± SEM
ASE aqueous	0.055	115.25 ± 6.25 ^a^
ASE MeOH	0.059	125.25 ± 1.25 ^a^
ASE EtOH	0.086	194.00 ± 5.00 ^b^
ASE EtOAc	0.065	141.50 ± 10.00 ^a^

Data expressed as mean ± SEM, *n* = 3. Data with different alphabet superscript letters show significant difference at *p* < 0.05, among different solvents, with multiple comparisons (one-way ANOVA, followed by Tukey’s test).

**Table 5:** IC_50_ values following treatment with mitragynine, doxorubicin, *M. speciosa* ASE leaf extracts in HEK-293 kidney cells and HeLa Chang liver cells.

Sample	IC_50_ Value	
	HEK-293 Kidney Cells	HeLa Chang Liver Cells
ASE aqueous extract	>500 µg/mL	>500 µg/mL
ASE MeOH extract	>500 µg/mL	>500 µg/mL
ASE EtOH extract	>500 µg/mL	>500 µg/mL
ASE EtOAc extract	>500 µg/mL	153.75 ± 31.75 µg/mL
Mitragynine	112.30 ± 17.59 µM	210.04 ± 0.80 µM
Doxorubicin ^a^	80.82 ± 12.05 µM	86.23 ± 27.49 µM

Note: Each value of IC_50_ represented mean ± SEM of three independent experiments.^a^ Positive control substance.

**Table 6:** MS/MS data of compounds identified tentatively in *M. speciosa* ASE aqueous, MeOH, EtOH, and EtOAc leaf extracts using UHPLC-ESI-QTOF-MS/MS.

Identification	Calculated m/z [M+H]+	Precursor ion Experimental m/z [M+H]+	Elemental Composition	Major Ions in MS/MS Spectra (Key Fragment Ions)	ASE Aqueous RT, min	ASEMeOH RT, min	ASE EtOH RT, min	ASE EtOAc RT, min	Ref.
Chlorogenic acid	355.1014	355.1023	C_16_H_18_O_9_	195.0649, 163.0393, 135.0445	2.99	-	2.49	2.39	[16]
Umbelliferone	163.0390	163.0394	C_9_H_6_O_3_	145.0280, 135.0445, 117.0340	3.00	3.25	3.06	3.09	[17]
*O*-coumaric acid	163.0427	165.0553	C_9_H_8_O_3_	147.0439, 109.0644, 165.0550, 121.0652	-	3.93	4.17	4.20	[16]
Quercetin 3-galactoside 7-rhamnoside	611.1607	611.1619	C_27_H_30_O_16_	465.1024, 449.1070, 303.0500	4.85	4.84	4.86	4.86	[16]
Rutin	611.1602	611.1621	C_27_H_30_O_16_	465.1022, 449.1072, 303.0496	5.01	5.03	5.03	5.03	[16]
Quercetin	303.0508	303.0509	C_15_H_10_O_7_	285.0395, 229.0496, 153.0183	-	5.26	5.30	5.25	[18]
Isoquercitrin	465.1028	465.1036	C_21_H_20_O_12_	303.0499, 153.0179	-	-	-	5.29	[19]
Vincamine	355.2016	355.2023	C_21_H_26_N_2_O_3_	338.1942, 224.1290, 144.0809	5.41	5.48	5.47	5.44	[20]
Rhynchophylline	385.2122	385.2122	C_22_H_28_N_2_O_4_	160.0759, 110.0965, 129.0544	-	5.87	5.93	5.89	[21]
Corynoxine B	385.2122	385.2132	C_22_H_28_N_2_O_4_	241.1338, 160.0758, 110.0964	6.55	6.44	6.44	6.44	[22]
Corynoxine	385.2122	385.2132	C_22_H_28_N_2_O_4_	353.1851, 160.0758, 110.0966	-	6.65	6.65	6.65	[22]
7-hydroxymitragynine	415.2227	415.2239	C_23_H_30_N_2_O_5_	400.1984, 190.0863, 110.0965	6.83	6.74	6.79	6.79	[22]
Mitragynine	399.2278	399.2278	C_23_H_30_N_2_O_4_	238.1440, 226.1441, 174.0913, 110.0967	7.44	7.34	7.34	7.49	[22]
Corynantheidine	369.2170	369.2173	C_22_H_28_ N_2_O_3_	238.1441, 226.1441, 110.0963	-	-	-	7.73	[22]
Speciogynine	399.2278	399.2302	C_23_H_30_N_2_O_4_	238.1443, 226.1445, 174.0916, 110.0965	-	-	7.74	7.79	[22]
Paynantheine	397.2122	397.2133	C_23_H_28_N_2_O_4_	236.1287, 224.1288, 174.0916, 159.0682	8.54	-	8.23	8.29	[22]
3-isopaynantheine	397.2122	397.2131	C_23_H_28_N_2_O_4_	200.1071, 174.0914, 159.0681	9.20	-	9.18	9.24	[22]
Speciociliatine	399.2278	399.2283	C_23_H_30_N_2_O_4_	238.1437, 226.1443, 174.0911, 110.0962	-	11.49	11.43	11.43	[22]
α-linolenic acid	279.2319	279.2325	C_18_H_30_O_2_	277.2318, 223.1688, 137.1329	-	-	13.33	13.48	[16]

## Data Availability

Not applicable.

## Supplementary Materials

Figure S1: HPLC-UV representatives overlap chromatograms of (a) blank, (b) mitragynine, (c) ASE aqueous extract, (d) ASE MeOH extract, (e) ASE EtOH extract, and (f) ASE EtOAc extract, Table S1: Percentage of cell viability of HEK-293 kidney cells against various concentrations of mitragynine, doxorubicin, and *M. speciosa* ASE leaf extracts, Table S2: Percentage of cell viability of HeLa Chang liver cells against various concentrations of mitragynine, doxorubicin, and *M. speciosa* ASE leaf extracts, Table S3: Effects of vehicle, morphine, and ASE extracts (aqueous, MeOH, EtOH, EtOAc) of *M. speciosa* leaves on nociceptive response in the tail-flick test, Table S4: Effects of vehicle, morphine and ASE extracts (aqueous, MeOH, EtOH, EtOAc) of *M. speciosa* leaves on nociceptive response in the hot plate test, Figure S2: Flow chart of research, Figure S3: EIMS mass spectrum of isolated mitragynine, Figure S4: ^1^H-NMR full spectrum (700 MHz, acetone-*d_6_*) of isolated mitragynine, Figure S5: ^13^C-NMR spectrum (175 MHz, acetone-*d_6_*) of isolated mitragynine, Table S5: 1H-NMR (700 MHz) and 13C-NMR (175 MHz) in acetone-d6 for mitragynine.

## Sample Availability

Samples of the compounds are not available from the authors.
