# Supplementary material for: Accelerated Solvent Extractions (ASE) of Mitragyna speciosa Korth. (Kratom) Leaves: Evaluation of Its Cytotoxicity and Antinociceptive Activity
Source: Molecules. 2021 Jun 17;26(12):3704. doi: 10.3390/molecules26123704 (PMC8234130; doi:10.3390/molecules26123704)
Supplement: Supplementary file 1 [file molecules-26-03704-s001.zip › molecules-1258763-supplementary.pdf]

molecules

MDPI

Supplementary Material

Accelerated Solvent Extractions (ASE) of *Mitragyna speciosa* Korth. (Kratom) Leaves: Evaluation of its Cytotoxicity and Antinociceptive Activity

Yong Sean Goh [1], Thiruventhan Karunakaran [1,2,] *, Vikneswaran Murugaiyah [3], Rameshkumar Santhanam [4], Mohamad Hafizi Abu Bakar [5] and Surash Ramanathan [1]

1 Centre for Drug Research, Universiti Sains Malaysia, 11800 USM, Pulau Pinang, Malaysia
2 School of Chemical Sciences, Universiti Sains Malaysia, 11800 USM, Pulau Pinang, Malaysia
3 School of Pharmaceutical Sciences, Universiti Sains Malaysia, 11800 USM, Pulau Pinang, Malaysia
4 BioSES Research Interest Group, Faculty of Science and Marine Environment, Universiti Malaysia Terengganu, 21030 Kuala Nerus, Malaysia
5 Bioprocess Technology Division, School of Industrial Technology, Universiti Sains Malaysia, 11800 USM, Penang, Malaysia
* Correspondence: thiruventhan@usm.my; Tel: +604-6533287

Abstract: *Mitragyna speciosa* Korth (kratom) is known for its psychoactive and analgesic properties. Mitragynine is the primary constituent present in kratom leaves. This study highlights the utilization of the green accelerated solvent extraction technique to produce a better, non-toxic and antinociceptive active botanical extract of kratom. ASE *M. speciosa* extract had a dry yield (0.53 – 2.91 g) and showed a constant mitragynine content (6.53 – 7.19 %) when extracted with organic solvents of different polarities. It only requires a shorter extraction time (5 minutes) and a reduced amount of solvents (less than 100 mL). A substantial amount of total phenolic (407.83 ± 2.50 GAE mg/g and flavonoids (194.00 ± 5.00 QE mg/g) were found in ASE kratom ethanol extract. The MTT test indicated that the ASE kratom ethanolic leaf extract is non-cytotoxic towards HEK-293 and HeLa Chang liver cells. In mice, ASE kratom ethanolic extract (200 mg/kg) demonstrated a better antinociceptive effect compared to methanol and ethyl acetate leaf extracts. The presence of bioactive indole alkaloids and flavonols such as mitragynine, paynantheine, quercetin and rutin in ASE kratom ethanolic leaf extract were detected using UHPLC-ESI-QTOF-MS/MS analysis supports its antinociceptive properties. ASE ethanolic leaf extract offers a better, safe, and cost-effective choice of test botanical extract for further preclinical studies.

Keywords: *Mitragyna speciosa;* Accelerated solvent extraction (ASE); Mitragynine; Cytotoxicity; Antinociceptive

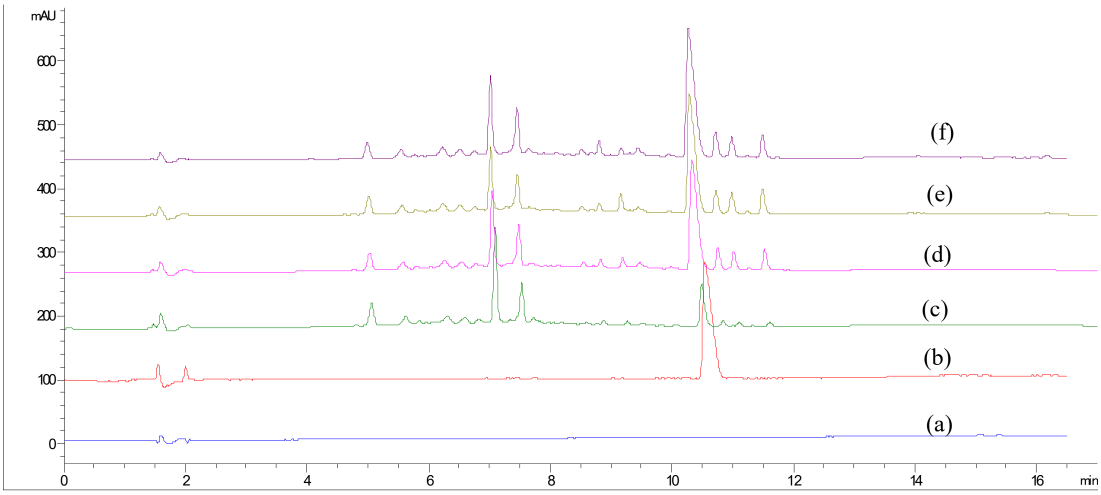

Figure 1. HPLC-UV representatives overlap chromatograms of (a) blank, (b) mitragynine, (c) ASE aqueous extract, (d) ASE MeOH extract, (e) ASE EtOH extract, and (f) ASE EtOAc extract. Chromatographic conditions: Agilent Zorbax Eclipse Plus C_{18} column (4.6 × 150 mm, 3.5 µm); mobile phase was 0.1 % formic acid and acetonitrile; flow rate was 1.0 mL/min; wavelength at 254 nm; injection concentration of 100 µg/mL and injection volume of 10 µL.

Table 1. Percentage of cell viability of HEK-293 kidney cells against various concentration of mitragynine, doxorubicin, and *M. speciosa* ASE leaf extracts.

**Sample**	**Concentration (µg/mL)**						
	**7.8125**	**15.625**	**31.25**	**62.5**	**125**	250	500
ASE aqueous extract	117.05±4.22	119.66±3.48	118.06±11.01*	130.28±11.90***	118.80±4.85	109.71±2.10	114.94±9.33*
ASE MeOH extract	96.31±6.17	97.14±8.68	94.41±5.92	80.70±2.46*	84.75±4.76	92.65±1.46	91.53±0.97
ASE EtOH extract	96.69±3.71	99.04±5.18	95.25±1.92	105.14±2.39	94.94±4.80	104.84±7.96	103.32±5.10
ASE EtOAc extract	110.12±6.63	105.51±2.12	94.79±1.81	103.14±5.53	101.44±1.96	111.89±3.58	101.02±2.29
Mitragynine	91.71± 1.45	80.66±0.95	70.09±0.63***	57.01±1.41***	41.30±0.95***	34.71±2.57***	28.60±1.88***
Doxorubicin	89.96±4.59	78.07±2.63	69.24±1.93***	52.78±0.44***	44.70±2.13***	38.24±3.60***	26.28±0.55***

Extracts diluted to 7.813 to 500 µg/mL. Data shown as mean ± SEM (n = 6). **p* < 0.05, ***p* < 0.01, ****p* <0.001 compared with DMSO blank (one-way ANOVA, followed by Dunnett's test).

Table 2. Percentage of cell viability of HeLa Chang liver cells against various concentration of mitragynine, doxorubicin, and *M. speciosa* ASE leaf extracts.

**Sample**	**Concentration (μg/mL)**						
	**7.8125**	**15.625**	**31.25**	**62.5**	**125**	250	500
ASE aqueous extract	95.85±2.53	93.46±1.97	89.53±2.27*	87.64±1.70**	85.14±1.89**	84.46±0.90***	60.52±4.19***
ASE MeOH extract	91.60±2.62	86.00±1.37*	84.07±0.17***	82.30±1.76***	77.86±0.81***	77.19±0.45***	63.25±1.89***
ASE EtOH extract	95.54±0.66	91.78±1.65	89.08±1.82*	80.13±2.08***	77.56±4.21***	57.84±4.28***	51.09±0.44***
ASE EtOAc extract	82.95± 0.71***	72.69±3.55***	66.39±5.29***	67.39±4.00***	58.93±2.15***	42.06±1.89***	42.51±1.48***
Mitragynine	83.94±0.49***	79.88±2.67***	77.49±1.49***	60.39±0.92***	39.89±1.13***	33.30±0.44***	27.32±1.02***
Doxorubicin	81.55±1.72***	77.17±2.52***	63.53±2.36***	50.66±2.96***	37.87±0.50***	33.86±1.19***	26.16±0.65***

Extracts diluted to 7.813 to 500 μg/mL. Data shown as mean ± SEM (n = 6). $^{*}p < 0.05$, $^{**}p < 0.01$, $^{***}p < 0.001$ compared with DMSO blank (one-way ANOVA, followed by Dunnett's test).

Table 3. Effects of vehicle, morphine and ASE extracts (aqueous, MeOH, EtOH, EtOAc) of *M. speciosa* leaves on nociceptive response in tail-flick test.

**Drug**	**Dose**	**Latency of nociceptive response (sec)**					
	**(mg/kg)**	T_{-30}	T_{0}	T_{30}	T_{60}	T_{90}	T_{120}
Vehicle	-	3.41 ± 0.40 ^{a}	4.59 ± 0.90 ^{a}	4.97 ± 0.79^{a}	5.50 ± 0.66^{a}	3.95 ± 0.27^{a}	4.19 ± 0.37^{a}
Morphine	5	5.03 ± 0.45 ^{a}	7.25 ± 0.81 ^{a}	10.10 ± 0.45^{**,b}	12.15 ± 0.46^{**,b}	11.72 ± 0.94^{***,b}	7.61 ± 0.86^{*,b}
ASE aqueous	200	3.90 ± 0.39 ^{a}	6.01 ± 0.66 ^{a}	9.54 ± 1.55^{a,b}	8.57 ± 1.68^{a,b}	5.05 ± 0.77^{a}	4.43 ± 0.60^{a}
ASE MeOH	200	4.75 ± 0.81 ^{a}	7.58 ± 0.40^{*,a}	10.26 ± 0.13^{***,b}	10.14 ± 1.42^{a,b}	8.21 ± 1.32^{*,a,b}	4.69 ± 0.81^{a,b}
ASE EtOH	200	5.60 ± 0.59 ^{a}	6.48 ± 0.98 ^{a}	9.68 ± 1.00^{**,b}	11.59 ± 1.10^{**,b}	8.58 ± 1.63^{*,a,b}	7.45 ± 0.43^{*,b}
ASE EtOAc	200	3.95 ± 0.76 ^{a}	5.40 ± 0.12 ^{a}	9.71 ± 1.31^{**,b}	9.59 ± 1.63^{a,b}	11.20 ± 1.15^{***,b}	5.58 ± 0.64^{a,b}

The drug was administrated orally (morphine injection, s.c.), nociceptive response was measured every 30 minutes over a 120-minutes period in mice. Data shown as mean ± SEM (n = 6). $^{*}p < 0.05$, $^{**}p < 0.01$, $^{***}p < 0.001$ compared with vehicle group (one-way ANOVA, followed by Dunnett's test). Data with different alphabet superscript letter show significant difference at $p < 0.05$, (one-way ANOVA, followed by multiple comparison, Tukey's test).

Table 4. Effects of vehicle, morphine and ASE extracts (aqueous, MeOH, EtOH, EtOAc) of *M. speciosa* leaves on nociceptive response in hot plate test.

**Drug**	**Dose**	**Latency of nociceptive response (sec)**					
	**(mg/kg)**	T_{-30}	T_{0}	T_{30}	T_{60}	T_{90}	T_{120}
Vehicle	-	3.38 ± 0.39 ^{a}	4.60 ± 0.55 ^{a}	4.49 ± 0.23^{a}	3.65 ± 0.45^{a}	4.06 ± 0.31^{a}	3.74 ± 0.28^{a}
Morphine	5	4.85 ± 0.74 ^{a}	4.91 ± 0.28 ^{a}	11.36 ± 1.45^{***,b}	11.57 ± 0.85^{***,b}	8.78 ± 1.47^{**,b}	8.16 ± 1.74^{**,b}
ASE aqueous	200	3.71 ± 0.30 ^{a}	4.36 ± 0.64 ^{a}	5.33 ± 0.59^{a}	5.34 ± 0.36^{a}	5.03 ± 0.52^{a,b}	3.79 ± 0.30^{a}
ASE MeOH	200	4.38 ± 0.33 ^{a}	4.75 ± 0.42 ^{a}	4.99 ± 0.68^{a}	7.39 ± 0.54^{**,a,b}	6.46 ± 0.87^{a,b}	4.31 ± 0.39^{a}
ASE EtOH	200	4.00 ± 0.33 ^{a}	5.81 ± 0.37 ^{a}	7.15 ± 1.00^{a}	8.88 ± 0.70^{***,b}	7.77 ± 0.46^{*,a,b}	5.17 ± 0.24^{a,b}
ASE EtOAc	200	3.71 ± 0.53 ^{a}	5.85 ± 1.27 ^{a}	7.20 ± 1.15^{a}	9.07 ± 0.95^{***,b}	7.52 ± 1.16^{*,a,b}	4.96 ± 0.64^{a,b}

The drug was administrated orally (morphine injection, s.c.), nociceptive response was measured every 30 minutes over a 120-minutes period in mice. Data shown as mean ± SEM (n = 6). $^{*}p < 0.05$, $^{**}p < 0.01$, $^{***}p < 0.001$ compared with vehicle group (one-way ANOVA, followed by Dunnett's test). Data with different alphabet superscript letter show significant difference at $p < 0.05$, (one-way ANOVA, followed by multiple comparison, Tukey's test).

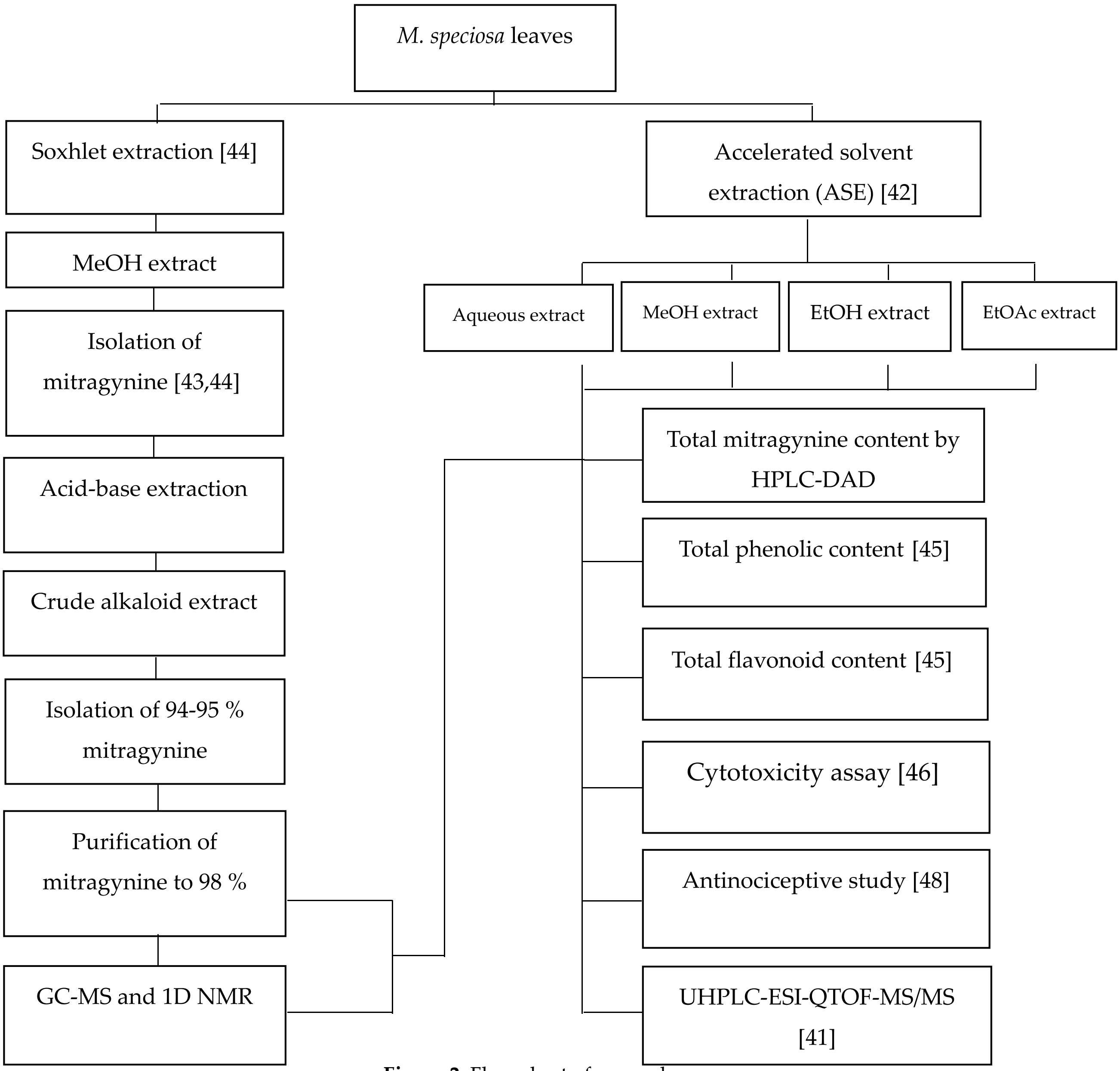

Figure 2. Flow chart of research.

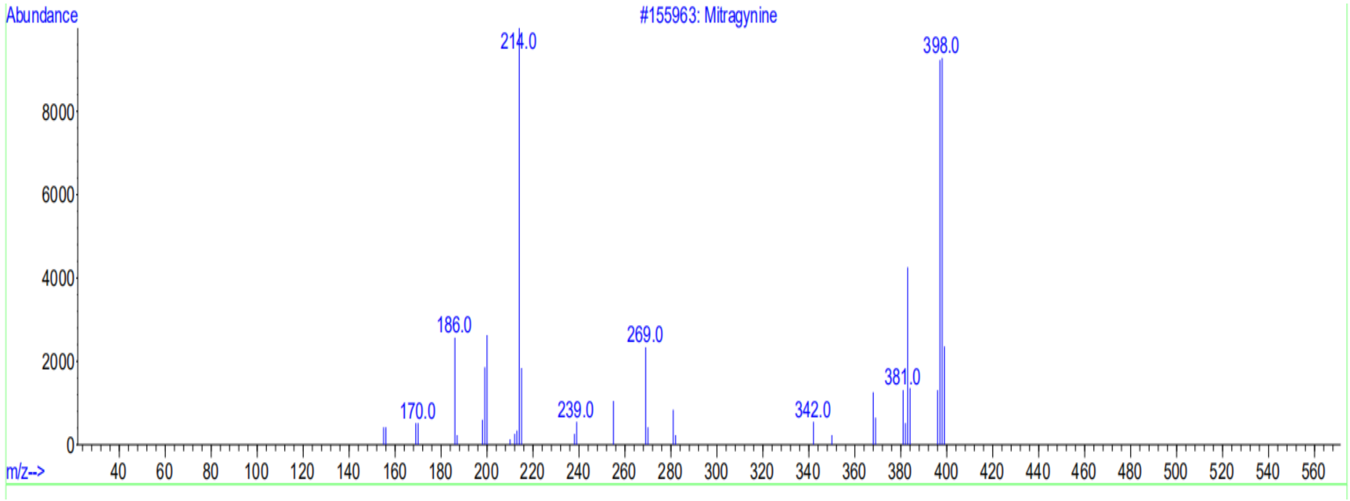

Figure 3. EIMS mass spectrum of isolated mitragynine.

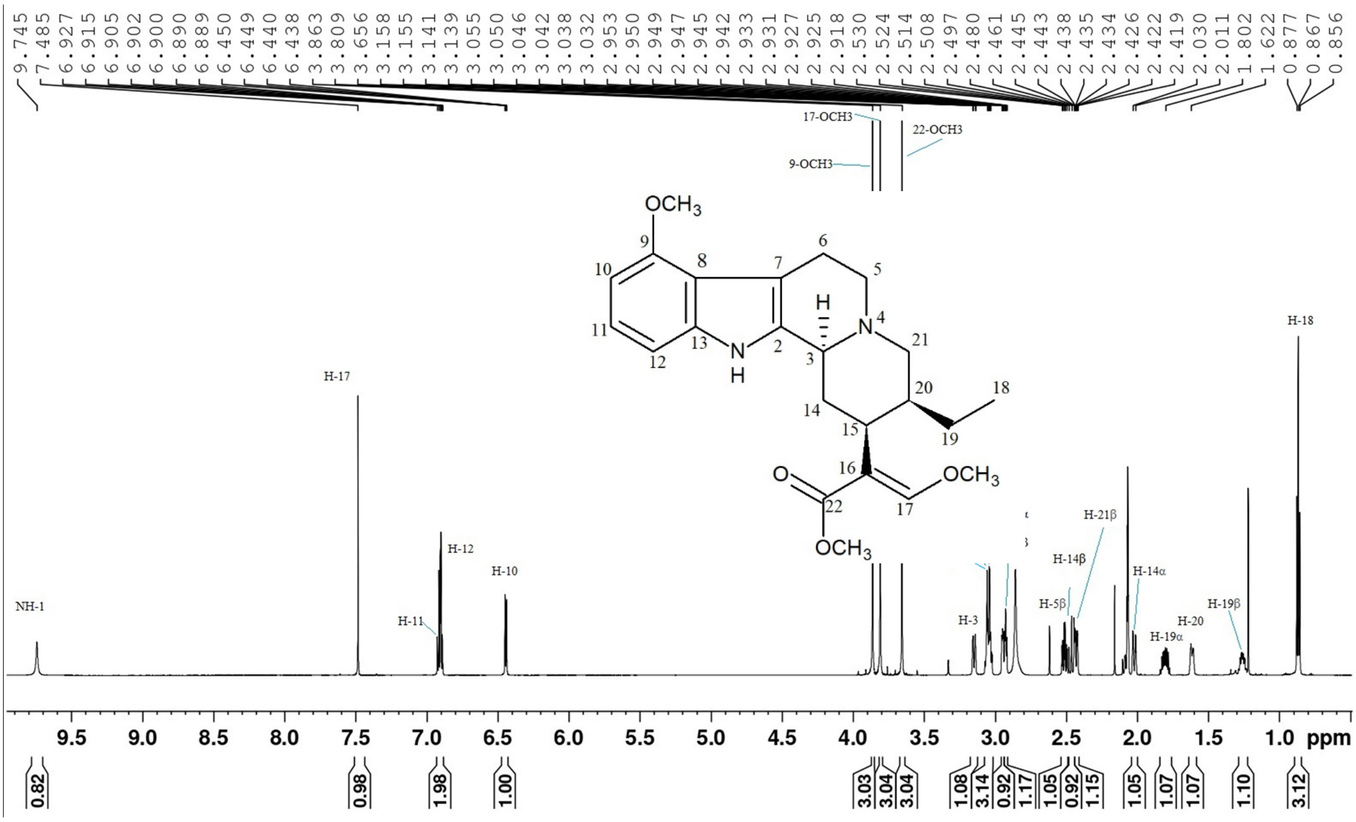

Figure 4. ^{1}H-NMR full spectrum (700 MHz, acetone-d_6) of isolated mitragynine.

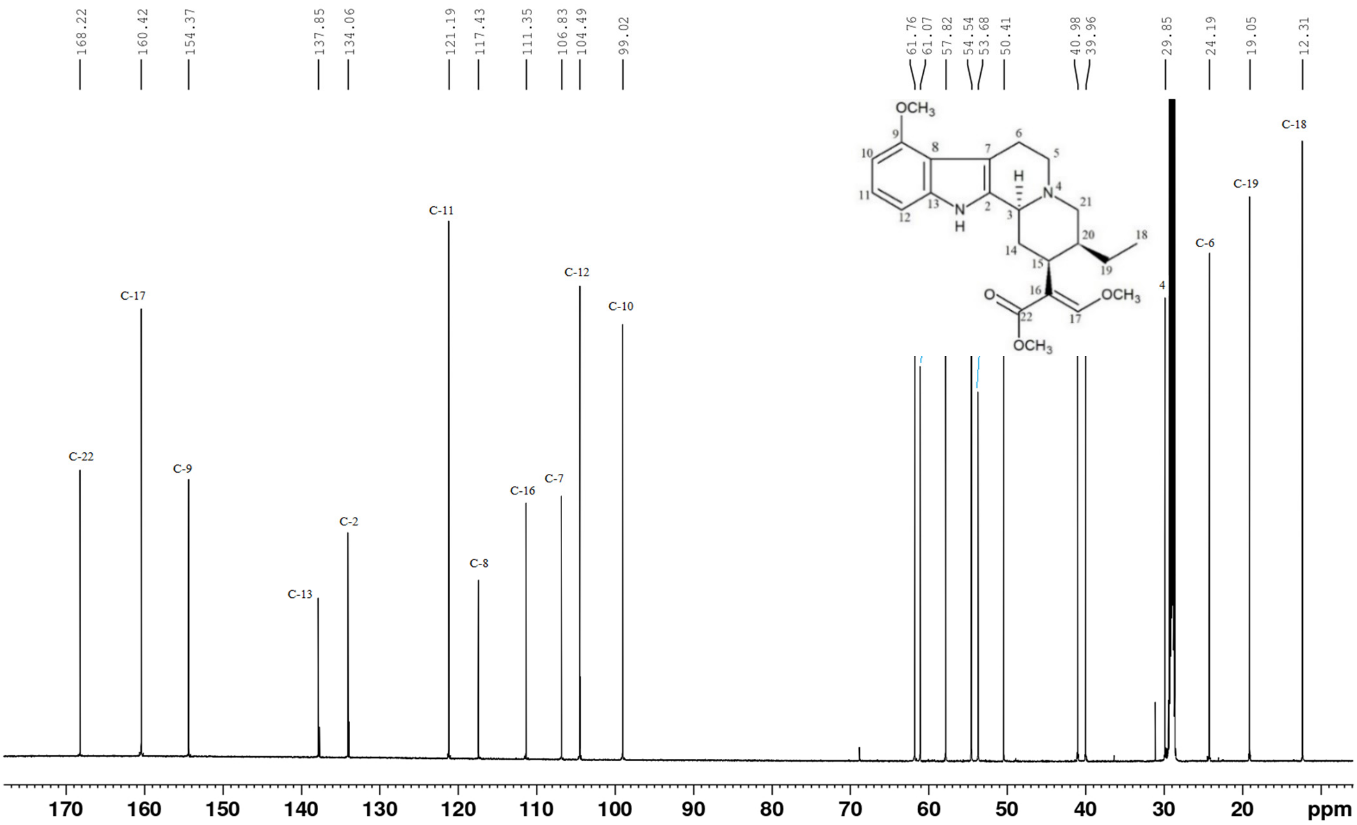

Figure 5. ^{13}C-NMR spectrum (175 MHz, acetone-d_6) of isolated mitragynine.

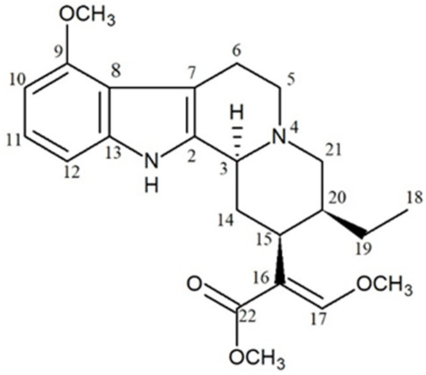

Table 5. ^{1}H-NMR (700 MHz) and ^{13}C-NMR (175 MHz) in acetone-d_6 for mitragynine.

**Position**	**^{1}H (δ)**	**^{13}C (δ)**
1	9.74 (s, 1H, NH)	-
2	-	134.1
3	3.15 (dd, 1H, J = 2.1 Hz & 11.9 Hz)	61.8
4	-	-
5	2.94 (br-dd, 1H, Hα),<br>2.52 (td, 1H, J = 4.2 Hz & 11.2 Hz, Hβ)	53.7
6	3.05 (m, 1H, Hα),<br>2.94 (br-t, 1H, Hβ)	24.2
7	-	106.8
8	-	117.4
9	-	154.4
10	6.44 (dd, 1H, J = 0.7 Hz & 7 Hz)	99.0
11	6.91 (t, 1H, J = 8.4 Hz)	121.2
12	6.90 (dd, 1H, J = 1.4 Hz & 7.7 Hz)	104.5
13	-	137.8
14	2.46 (br-t, 1H, Hβ)<br>2.02 (dt, 1H, J = 2.8 Hz & 16.1 Hz, Hα)	29.9
15	3.06 (m, 1H)	40.0
16	-	111.4
17	7.48 (s, 1H)	160.4
18	0.87 (t, 3H, J = 7.0 Hz)	12.3
19	1.80 (m, 1H, Hα),<br>1.26 (m, 1H, Hβ)	19.1
20	1.62 (br-d, 1H)	41.0
21	3.07 (m, 1H, Hα),<br>2.44 – 2.41 (dt, 1H, Hβ)	57.8
22	-	168.2
9 -OCH_3	3.86 (s, 3H)	54.5
17-OCH_3	3.81 (s, 3H)	61.1
22 -OCH_3	3.66 (s, 3H)	50.4

The ^{1}H-NMR and ^{13}C-NMR spectral data are consistent with the published data [43].
